# Diversity of *Bradyrhizobium* in Non-Leguminous Sorghum Plants: *B. ottawaense* Isolates Unique in Genes for N_2_O Reductase and Lack of the Type VI Secretion System

**DOI:** 10.1264/jsme2.ME19102

**Published:** 2020-01-11

**Authors:** Sawa Wasai-Hara, Shintaro Hara, Takashi Morikawa, Masayuki Sugawara, Hideto Takami, Junich Yoneda, Tsuyoshi Tokunaga, Kiwamu Minamisawa

**Affiliations:** 1 Graduate School of Life Sciences, Tohoku University, Katahira, Aoba-ku, Sendai 980–8577, Japan; 2 Yokohama Institute, Japan Agency for Marine-Earth Science and Technology (JAMSTEC), Shouwa-machi, Kanazawa, Yokohama 236–0001, Japan; 3 Earthnote Co., Ltd., Isagawa, Nago, Okinawa, 905–1152, Japan

**Keywords:** *Bradyrhizobium*, sorghum, nitrogen fixation, N_2_O reductase gene, protein secretion system

## Abstract

Diverse members of *Bradyrhizobium diazoefficiens*, *B. japonicum*, and *B. ottawaense* were isolated from the roots of field-grown sorghum plants in Fukushima, and classified into “Rhizobia” with nodulated soybeans, “Free-living diazotrophs”, and “Non-diazotrophs” by nitrogen fixation and nodulation assays. Genome analyses revealed that *B. ottawaense* members possessed genes for N_2_O reduction, but lacked those for the Type VI secretion system (T6SS). T6SS is a new bacterial weapon against microbial competitors. Since T6SS-possessing *B. diazoefficiens* and *B. japonicum* have mainly been isolated from soybean nodules in Japan, T6SS-lacking *B. ottawaense* members may be a cryptic lineage of soybean bradyrhizobia in Japan.

*Bradyrhizobium* was initially identified as a symbiotic N_2_-fixing bacterium in leguminous plants (Jordan, 1982). However, non-symbiotic and/or non-diazotrophic bradyrhizobia have frequently been found in the soil (Van Insberghe *et al.*, 2015; Jones *et al.*, 2016) and roots of non-leguminous plants (Schneijderberg *et al.*, 2018). In the soil of North American forests (Van Insberghe *et al.*, 2015), dominant *Bradyrhizobium* lacks symbiosis islands on the genome, including *nif* and *nod* genes, for N_2_ fixation and nodulation, respectively. Recent phylogenomic studies revealed that *Bradyrhizobium* includes species with diverse lifestyle traits, including N_2_ fixation, nodulation, and photosynthesis, across lineages (Avontuur *et al.*, 2019; Ormeño-Orrillo and Martínez-Romero, 2019).

N_2_ fixation by endophytic *Bradyrhizobium* has been examined in non-leguminous crops for sustainable agriculture. *B. sacchari*, *Bradyrhizobium* sp. AT1, and *Bradyrhizobium* sp. SUTN9-2 were identified as diazotrophic endophytes in sugarcane (Rouws *et al.*, 2014), sweet potato (Terakado-Tonooka *et al.*, 2013), and rice (Greetatorn *et al.*, 2019), respectively. Hara *et al.* (2019) showed that the functional N_2_-fixing bradyrhizobia (TM122 and TM124) in sorghum roots were phylogenetically close to photosynthetic *B. oligotrophicum* S58^T^ (Okubo *et al.*, 2013) and non-nodulating *Bradyrhizobium* sp. S23321 (Okubo *et al.*, 2012). The *nif* genes of “Free-living diazotrophs” TM122, TM124, S58^T^, and S23321 are markedly different from those on the symbiosis islands of nodule-forming *Bradyrhizobium* (“Rhizobia”: *B. diazoefficiens* and *B. japonicum*) with respect to the G+C content of the *nifDK* genes (Okubo *et al.*, 2016), *nifV* (Hara *et al.*, 2019), and possibly *nif* gene regulation (Dixon and Kahn, 2004), which have been overlooked in previous phylogenetic studies targeting the *nifH* gene alone.

The aims of the present study were (i) to examine the abilities for free-living N_2_ fixation and nodulation by *Bradyrhizobium* isolates that inhabit the roots of non-leguminous sorghum plants and (ii) to investigate whether the isolates have other functional differences via a genome analysis. To obtain diverse sorghum bradyrhizobia, in addition to isolates by direct isolation described previously (Hara *et al.*, 2019), we applied the legume trapping method (soybean nodulation) using the roots of sorghum obtained in a previous study (Hara *et al.*, 2019).

The roots of the sorghum line KM2 (102 d after transplant) stored at –80°C (Hara *et al.*, 2019) were surface-sterilized with 2.5% NaOCl at room temperature for 10 min, and washed ten times with sterilized water. Approximately 30 g of the root tissues were powdered with liquid N_2_ using a mortar and pestle and 200 mL of 50‍ ‍mM Tris-HCl buffer (pH 7.5) was thoroughly mixed and passed through a Miracloth (Millipore) to remove plant residues. Filtered samples were centrifuged at 9,876×*g* for 10 min. The pellet was suspended in 10 mL of 50‍ ‍mM Tris-HCl buffer (pH 7.5), which was then inoculated into surface-sterilized seeds of the soybean cultivar Enrei in a Leonard jar assembly (Inaba *et al.*, 2012). After growing the soybean plants at 23°C for 3‍ ‍weeks, the bacteroid cells of the nodule section were streaked on 1/100 strength NA agar medium (Difco^TM^ Nutrient Broth, Becton, Dickinson and Company). After 10‍ ‍d of incubation at 28°C, bacterial colonies were further purified twice by single colony isolation. Their 16S–23S rRNA gene internal transcribed spacer (ITS) sequences were elucidated to examine whether the isolates belonged to the genus *Bradyrhizobium* (Willems *et al.*, 2003; Saeki *et al.*, 2013; Shiina *et al.*, 2014).

Acetylene reduction activity (ARA) under free-living conditions was evaluated as described previously (Hara *et al.*, 2019). Briefly, isolates were pre-cultured in HM broth medium and inoculated into test tubes containing Rennie semi-solid medium (Rennie, 1981). After 3 d of cultivation, acetylene was introduced at a final concentration of 10% (v/v) in the headspace of the test tube. The resultant ethylene concentration was assessed by gas chromatography (Hara *et al.*, 2019). Nodulation was evaluated by performing inoculation tests on three leguminous plants: soybean (*Glycine max* cv. Enrei), cowpea (*Vigna unguiculata* cv. California black eye), and siratro (*Macroptilium atropurpureum*). *Bradyrhizobium* cells were inoculated into the surface-sterilized seeds of the three plants (10^7^ cells seed^–1^) (Hara *et al.*, 2019). Root nodulation and plant growth were observed after cultivation under a daily light cycle of 16 h of light and 8 h of dark at 25°C for 3 (soybean) or 5 (cowpea and siratro) weeks in a growth cabinet (Koito Electric Industries).

DNA was extracted from bacterial isolates using the Illustra^TM^ Bacteria Genomic Prep Mini Spin kit (GE Healthcare). Draft genome sequences were elucidated using MiSeq (Hara *et al.*, 2019). To analyze phylogenic relationships, 31 single-copy genes were extracted from the draft genome using AMPHORA2 (Wu and Scott, 2012). A phylogenic tree was constructed based on the concatenated amphora gene by MEGA v. 7.0 (Tamura *et al.*, 2011) and the neighbor-joining method (Saitou and Nei, 1987). The completion patterns of the KEGG modules for metabolic and physiological functions were examined using the new MAPLE system v. 2.3.1 (Takami *et al.*, 2016; Arai *et al.*, 2018). We used GHOSTX as a homology search engine because it is markedly faster than BLAST (Arai *et al.*, 2018). The total DNAs of SG09 and TM102 were extracted as described previously (Minamisawa, 1990; Minamisawa *et al.*, 1992; Rouws *et al.*, 2014) to obtain high quality samples for a complete genome analysis. Complete genomes were elucidated using the PacBio RSII (Pacific Biosciences) platform. Island viewer4 based on the SIGI-HMM and IslandPath-DIMOB programs was used to detect genomic islands (Bertelli *et al.*, 2017). The comparison of gene clusters and bl2seq was performed using GenomeMatcher (Ohtsubo *et al.*, 2008).

We obtained 38 *Bradyrhizobium* isolates from different nodules of 20 soybean plants inoculated with the macerate of the surface-sterilized roots of field-grown sorghum, termed the “Trapping” method (Table 1 and S1). We also used 7 bradyrhizobial isolates in oligotrophic agar media from the same plant materials of field-grown sorghum roots as described previously (Hara *et al.*, 2019) (Table 1 and S1). Based on the ITS sequence, 45 isolates from sorghum roots were grouped into 6 operational taxonomic units (OTUs), which were phylogenetically close to *B. diazoefficiens*, *B. japonicum*, *B. ottawaense*, and *Bradyrhizobium* sp. S23321 (Fig. S1).

Soybean bradyrhizobia (“Rhizobia”) often do not exhibit any N_2_-fixing activity under free-living conditions, but show symbiotic N_2_ fixation in nodule bacteroids (Kuykendall, 2005), whereas diazotrophic bacteria from non-leguminous plants and soils (“Free-living diazotrophs”) exhibit N_2_-fixing activity under free-living conditions (Okubo *et al.*, 2012; Okubo *et al.*, 2013; Terakado-Tonooka *et al.*, 2013; Rouws *et al.*, 2014; de Matos *et al.*, 2017; Hara *et al.*, 2019). To test the differential N_2_-fixing capability between “Rhizobia” and “Free-living diazotrophs”, the ARA of the reference strains were assessed in Rennie semi-solid medium. ARA (<0.01 nmol C_2_H_4_ h^–1^ tube^–1^) was not detected in the culture of the soybean bradyrhizobia of *B. diazoefficiens* USDA110^T^ or *B. japonicum* USDA 6^T^, irrespective of pellicle formation by cell growth (Fig. S2A and B). This is most likely due to the rhizobial *nif* gene cluster lacking *nifV*—an essential gene for the production of homocitrate, a necessary component of the FeMo cofactor present in nitrogenase—which is found in “Free-living diazotrophs” (Hakoyama *et al.*, 2009; Okubo *et al.*, 2016; Hara *et al.*, 2019). In contrast, significant ARA (5–33 nmol C_2_H_4_ h^–1^ tube^–1^) was observed in the “Free-living diazotrophs” of *B. oligotrophicum* S58^T^, *Bradyrhizobium* sp. S23321, and *Bradyrhizobium* sp. BTAi1 (Fig. S2C, D, and E). This result confirmed the above criteria of N_2_-fixing activity between “Rhizobia” and “Free-living diazotrophs” under free-living conditions (Kuykendall, 2005). Thus, we adopted this method for the 45 isolates (Table 1 and S1). ARA was not observed among the 38 isolates with prefixes SF, SG, and SH by the trapping method or the 4 isolates obtained by direct isolation from sorghum roots (TM220, TM102, TM233, and TM239) (Table 1 and S1), whereas ARA was detected in TM221 under free-living conditions (Fig. S2F), in addition to previously reported TM122 and TM124 (Hara *et al.*, 2019).

TM220 nodulated the leguminous plants of soybean, cowpea, and siratro, whereas the other 6 isolates (TM102, TM122, TM124, TM221, TM233, and TM239) did not (Fig. S3). The ARA of the soybean nodules formed by TM220 was 24.3 μmol h^–1^ plant^–1^ (Fig. S3), suggesting that TM220 fixed N_2_ symbiotically and fell into the category of “Rhizobia” (Table 1 and S1).

Based on the ARA and nodulation assays, 45 isolates of sorghum bradyrhizobia were categorized into “Rhizobia”, “Free-living diazotrophs”, or “Non-diazotrophs”: “Rhizobia” showed nodulation and symbiotic N_2_ fixation abilities, but no N_2_ fixation in free-living cells. “Free-living diazotrophs” lacked the ability to nodulate legumes, but possessed the capability to fix N_2_ under free-living conditions. “Non-diazotrophs” lacked any potential to fix nitrogen or for nodulation (Table 1).

Draft genomes were elucidated for 16 representative isolates, including 6 OTUs (Table S1, Fig. S1) from the 38 nodule isolates and 4 direct isolates (TM220, TM221, TM233, and TM239) (Hara *et al.*, 2019). According to the phylogenetic relationships obtained using AMPHORA, we defined three major taxonomic groups: groups D, J, and W, which corresponded to *B. diazoefficiens*, *B. japonicum*, and *B. ottawaense*, respectively (Fig. 1A). “Rhizobia” with the prefixes SF/SG/SH belonged to group D, J, or W. “Free-living diazotrophs” TM221, TM124, and TM122 were not included in group D, J, or W. The non-diazotrophic isolates TM102, TM233, and TM239 fell exclusively into the group W. Thus, group W members included “Non-diazotrophs” (TM102, TM233, and TM239) and “Rhizobia” (SG09 and SG11), which contained a new species, *B. ottawaense*, of soybean bradyrhizobia in Canada (Yu *et al.*, 2014; Nguyen *et al.*, 2018). In agricultural fields in Japan, the major soybean bradyrhizobia consists of the groups D (*B. diazoefficiens*), J (*B. japonicam*), and *B. elkanii* (Saeki *et al.*, 2013; Shiina *et al.*, 2014). Therefore, SG09 and SG11 of group W (*B. ottawaense*) were likely cryptic soybean bradyrhizobia in Japan, which were initially isolated by the trapping method from the roots of field-grown sorghum plants (Fig. 1). These results also demonstrated that diverse *Bradyrhizobium* species, including “Rhizobia”, “Free-living diazotrophs”, and “Non-diazotrophs”, were simultaneously inhabiting the roots of a single line of field-grown sorghum plants.

Based on these draft genomes, we examined differences in the gene repertories responsible for these phenotypes and other functions. Genes for N_2_ fixation were detected in the sorghum isolates of “Rhizobia” and “Free-living diazotrophs,” including type strains, such as USDA110, USDA6, OO99, and S58, while they were not detected in the “Non-diazotrophs” of sorghum isolates (Fig. 1B). In addition, “Rhizobia” exclusively possessed the genes for nodulation, except for *B. oligotrophicum* S58 (Fig. 1B). These results supported the validity of our functional prediction obtained using the MAPLE system.

The co-existence of “Rhizobia” and “Non-diazotrophs” within the group W (Fig. 1A) prompted us to investigate their genome architecture, including their symbiosis islands. The SG09 (“Rhizobia”) genome was a single circular chromosome (8.44 Mb) with a typical symbiosis island (759 kbp) adjacent to the Val-tRNA gene (Fig. S4, Table S2), which is a target gene for symbiosis island transfer in soybean bradyrhizobia (Kaneko *et al.*, 2002; Kaneko *et al.*, 2011). On the other hand, TM102 (“Non-diazotrophs”) completely lacked symbiosis islands, including *nif* and *nod* gene clusters, on their single circular chromosome (7.36‍ ‍Mb) (Fig. S4). When MiSeq reads were mapped on the SG09 genome, TM102, TM223, and TM239 (“Non-diazotrophs”) apparently lacked symbiosis islands, including *nif/nod* genes, while SG11 and SG09 (“Rhizobia”) conserved their symbiosis island (Fig. S5). Thus, the existence of symbiosis islands delineated “Rhizobia” and “Non-diazotrophs” on the genomes of group W members (Fig. 1A). The horizontal gene transfer of symbiosis islands may occur within group W rather than in groups D and J (Barcellos *et al.*, 2007; Andrews *et al.*, 2018, Wasai and Minamisawa, 2018).

Denitrification genes were consistently detected in members of groups D, J, and W (Fig. 1B). Denitrification generally requires four enzymes: nitrate reductase (Nap), nitrite reductase (Nir), nitric oxide reductase (Nor), and N_2_O reductase (Nos) (Zumft, 1997; Jang *et al.*, 2018; Sánchez *et al.*, 2019). An examination of the genes of these four enzymes indicated that *nos* gene clusters encoding N_2_O reductase were consistently found in group D and W members (black circle in Fig. 1B and S5). Although soybean bradyrhizobia carrying *nosRZDYFLX* genes are able to mitigate the emission of N_2_O, a greenhouse gas, from soil (Itakura *et al.*, 2013; Akiyama *et al.*, 2016; Saeki *et al.*, 2017), the existence of *nos* genes appears to be confined within *B. diazoefficiens* (group D) in soybean bradyrhizobia (Itakura *et al.*, 2013; Shiina *et al.*, 2014; Akiyama *et al.*, 2016; Saeki *et al.*, 2017). Thus, group W members may mitigate N_2_O emission from soil as well as *B. diazoefficiens*. Bradyrhizobial isolates phylogenetically close to *B. ottawaense* from African woody legumes were recently confirmed to reduce N_2_O to N_2_ (Mania *et al.*, 2019).

The existence of protein secretion systems was markedly different among the isolates or their lineages (Fig. 1B). Genes for the Type VI secretion system (T6SS) were found exclusively in members of groups D and J, whereas group W members completely lacked T6SS genes. Gene organization showed the typical *imp* gene cluster of T6SS (Fig. 1C), which is widespread in Gram-negative bacteria, including “Rhizobia,” and the contact-dependent apparatus related to inter-bacterial competition and bacterial interactions with eukaryotic cells (Bingle *et al.*, 2008; Records, 2011; Coulthurst, 2019). T6SS effectors and cognate immunity proteins exerted antibacterial and antifungal effects, which allowed the bacterium to compete with rival microbes and highlighted their roles within microbial communities; however, their roles in “Rhizobia” remain unclear (Bingle *et al.*, 2008; Records, 2011; Coulthurst, 2019). Since group W members have not been identified in soybean endosymbionts in agricultural environments in Japan (Saeki *et al.*, 2013; Shiina *et al.*, 2014), a hypothesis is that the T6SS-lacking members of group W may be less competitive than the T6SS-possessing members of groups D and J in agricultural environments in Japan (Saeki *et al.*, 2013; Shiina *et al.*, 2014).

The Type IV secretion system (T4SS) was detected in several isolates of “Rhizobia” and “Non-diazotroph” regardless of phylogenetic relationships (Fig. 1B). Most T4SS genes were annotated to the *trb* genes for conjugation (Wallden *et al.*, 2010; Sugawara *et al.*, 2013), while T4SS genes in SF01 were highly homologous to the *vir* genes for‍ ‍the T-DNA transfer of *Agrobacterium* (Wallden *et al.*, 2010; Sugawara *et al.*, 2013) and effector secretion of *Sinorhizobium* (Sugawara *et al.*, 2013) (SG01 in Fig. 1C).

The Type III secretion system (T3SS) was well conserved in all isolates and reference strains of “Rhizobia”, which corresponded to the conventional *rhc* gene cluster responsible for host specificities (SF01 in Fig. 1C) (Viprey *et al.*, 1998; Tsukui *et al.*, 2013; Tampakaki, 2014; Sugawara *et al.*, 2018). TM233 and TM239 (“Non-diazotrophs”) in group W possessed T3SS that resembled *ysc* genes encoding pathogenic T3SS of *Yersinia* species (TM233 and TM239 in Fig. 1C) (Viprey *et al.*, 1998; Cornelis, 2002; Tampakaki, 2014). To the best of our knowledge, this is the first example of pathogenic type T3SS genes existing in the genus *Bradyrhizobium*.

In conclusion, the usage of non-leguminous sorghum plants revealed the greater diversity of *Bradyrhizobium* than previously considered. Members of group W of *Bradyrhizobium* (*B. ottawaense*), with and without symbiosis islands, were indigenous to Japan. Relative to conventional soybean bradyrhizobia, their genomes possessed unique traits for the presence of *nos* genes and the absence of T6SS, related to their presumptive differences in competition and plant associations.

## Supplementary Material

Supplementary Material

## Figures and Tables

**Fig. 1.**
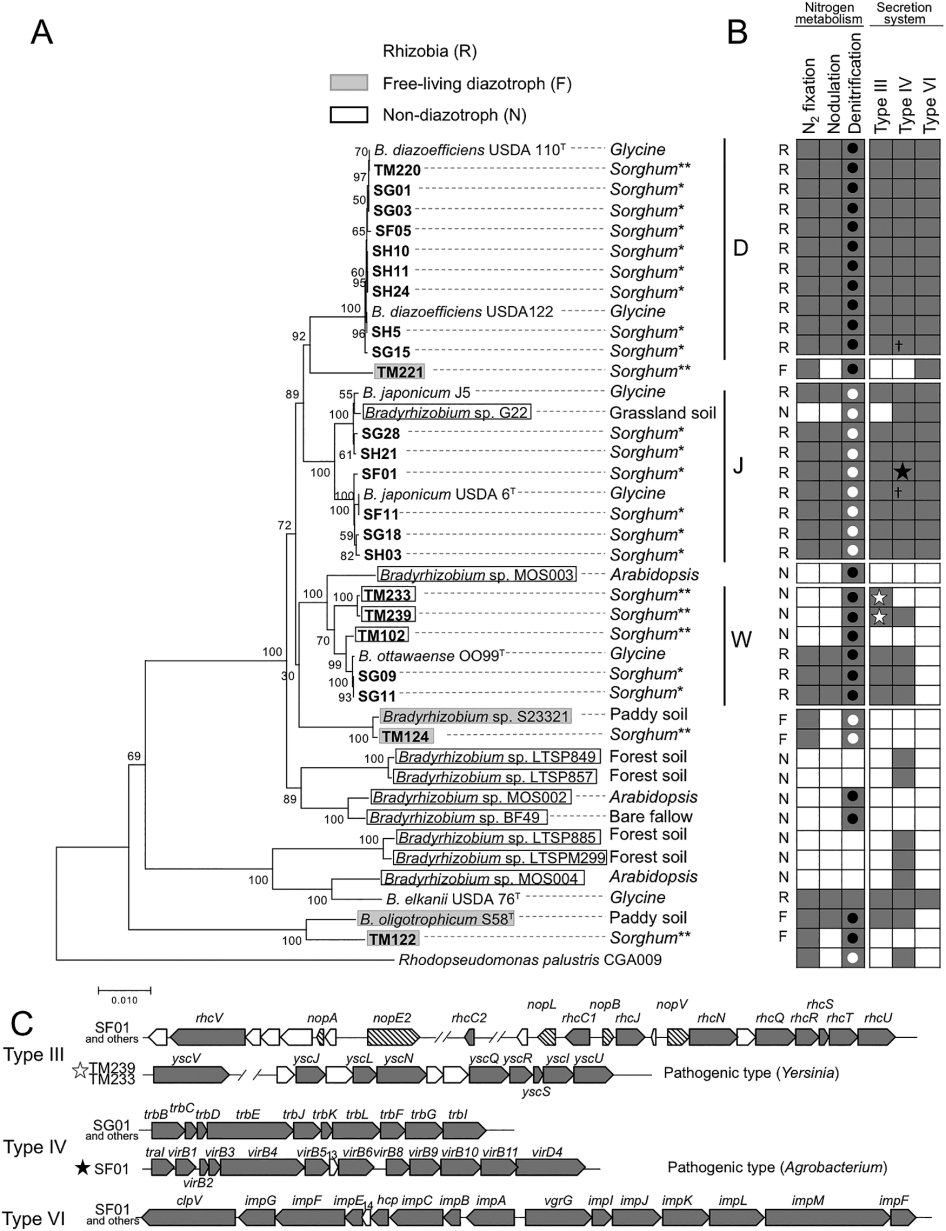
Phylogenetic relationship and functional potential of bradyrhizobial isolates from sorghum roots. (A) The phylogenetic relationship based on 31 single copies of AMPHORA housekeeping genes (Wu and Scott, 2012). The strains shaded in gray denote “Free-living diazotroph” (F). Strains framed with a black square denote “Non-diazotroph” (N). The other strains denote “Rhizobia” (R). The plant genera (italics) or soil types on the right-hand side shows the origins of the isolates. In sorghum isolates, the asterisk (*) and double asterisks (**) denote the isolates in the present study and those from previous isolates (Hara *et al.*, 2019), respectively. (B) Functional potentials of nitrogen fixation (M00175), nodulation (M00664), denitrification (M00529), and secretion systems (M00332, M00333, and M00334) evaluated by MAPLE v. 2.3.1 (Takami *et al.*, 2016; Arai *et al.*, 2018). The closed and open circles in the denitrification column denote the isolates possessing genes for the denitrification steps from nitrate to dinitrogen, and nitrate to nitrous oxide, respectively. ☆ in the Type III secretion system and ★ in the Type IV secretion system denote the isolates possessing different gene organizations from the other strains, which are described in detail in panel C. †in the Type IV secretion system shows that the gene cluster was partially conserved. (C) Detailed gene organization of the Type III (T3SS), IV (T4SS), and VI (T6SS) secretion systems of the isolates. Hypothetical genes were expressed as white arrows. The genomic positions of separate T3SS gene clusters were unknown due to different contigs by the draft genome assembly of MiSeq sequences.

**Table 1.** Categories of *Bradyrhizobium* isolates from sorghum roots based on free-living nitrogen fixation and legume nodulation.

Category*^a^*	Isolation method*^b^*	N_2_ fixation*^c^* (free-living)	Nodulation*^d^*	Tested	Isolate
“Rhizobia”	Trapping	–	+	38	Prefix of SF, SG, and SH
“Rhizobia”	Direct	–	+	1	TM220
“Free-living diazotrophs”	Direct	+	–	3	TM122, TM124, TM221
“Non-diazotrophs”	Direct	–	–	3	TM102, TM233, TM239

*^a^* “Rhizobia” and “Free-living diazotrophs” indicate the nodule-forming bacteria to legume plants and N_2_-fixing bacteria associated with non-leguminous plants, respectively. *^b^* “Trapping” indicates the soybean trapping method (see text), while “Direct” indicates the direct isolation of bradyrhizobia in oligotrophic agar media (Hara *et al.*, 2019). *^c^* N_2_-fixing activity was evaluated via an acetylene reduction assay under free-living conditions. *^d^* Nodulation ability was evaluated using an inoculation test with soybean, cowpea, and siratro. The isolates obtained from the soybean nodules of “Rhizobia” using the “Trapping” method were regarded as having a positive nodulation capability.
