# Supplementary material for: Diversity of Bradyrhizobium in Non-Leguminous Sorghum Plants: B. ottawaense Isolates Unique in Genes for N2O Reductase and Lack of the Type VI Secretion System
Source: Microbes Environ. 2020 Jan 11;35(1):ME19102. doi: 10.1264/jsme2.ME19102 (PMC7104290; doi:10.1264/jsme2.ME19102)
Supplement: Supplementary file 1 — Supplementary Material [file 35_19102_s1.pdf]

Supplementary figures

Diversity of *Bradyrhizobium* in Non-Leguminous Sorghum Plants: *B. ottawaense* Isolates Unique in Genes for N2O Reductase and Lack of the Type VI Secretion System

Sawa Wasai-Hara[1], Shintaro Hara[1], Takashi Morikawa[1], Masayuki Sugawara[1], Hideto Takami[2], Junich Yoneda[3], Tsuyoshi Tokunaga[3], and Kiwamu Minamisawa[1*]

[1]Graduate School of Life Sciences, Tohoku University, Katahira, Aoba-ku, Sendai 980-8577, Japan, [2]Yokohama Institute, Japan Agency for Marine-Earth Science and Technology (JAMSTEC), Shouwa-machi, Kanazawa, Yokohama 236-0001, Japan, [3]Earthnote Co., Ltd., Isagawa, Nago, Okinawa, 905-1152, Japan

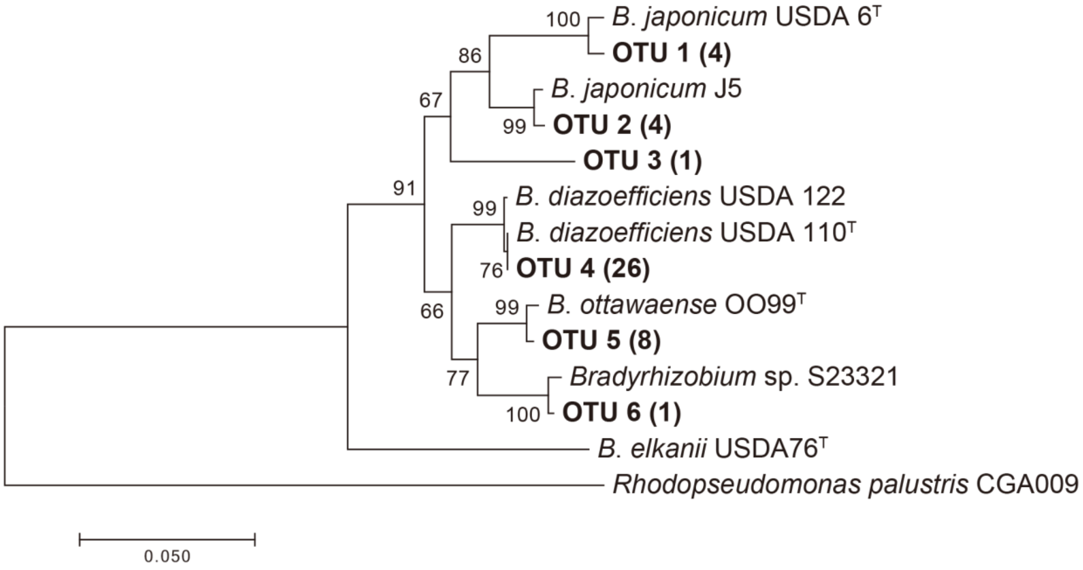

Fig. S1. Phylogenic tree of ITS sequence in sorghum bradyrhizobial isolates. The numbers in parentheses denote the number of isolates included in each OTU. As *B. oligtrophicum* S58^T and TM122 showed irregular ITS sequences that varied from those of the OTU 1~OTU 6 members, they were excluded from this phylogenetic tree. However, *B. oligotrophicum* S58^T and TM122 were included in the subsequent analysis (Fig. 1).

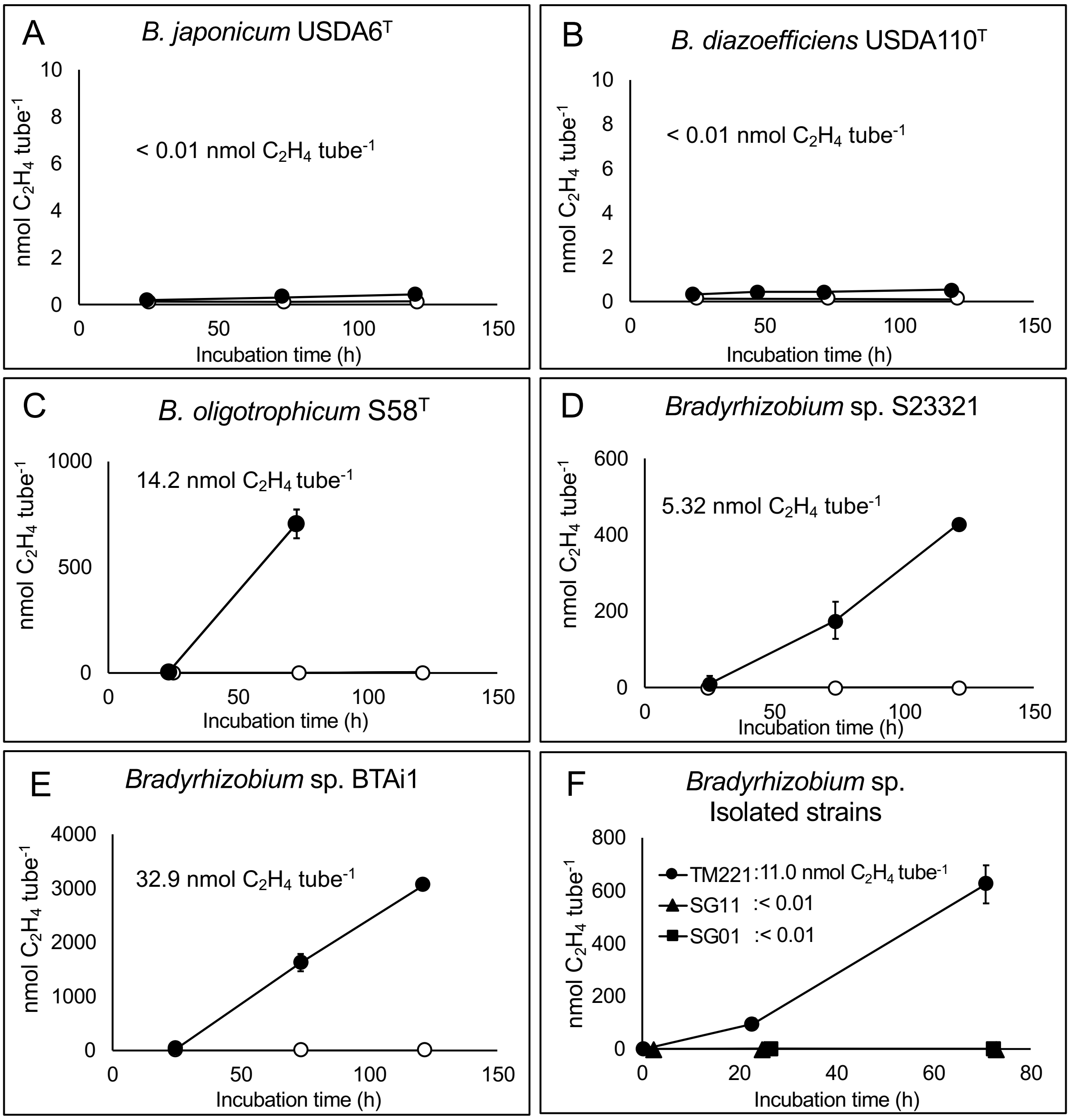

Fig. S2. Acetylene-reducing activity in *Bradyrhizobium japonicum* USDA 6^T, *B. diazoefficiens* USDA 110^T, *B. oligotrophicum* S58^T, *Bradyrhizobium* sp. S23321, *Bradyrhizobium* sp. BTAi1, and *Bradyrhizobium* sp. TM221, SG01, and SG11 in the cultures of Rennie semi-solid medium. The x-axis shows the incubation time after the addition of 10% (v/v) acetylene, while the y-axis shows the amount of ethylene in the headspace of the test tube. The black and white symbols denoted the ARA in the inoculated and uninoculated test tubes. The values represent the mean and SD (bar) from triplicate experiments.

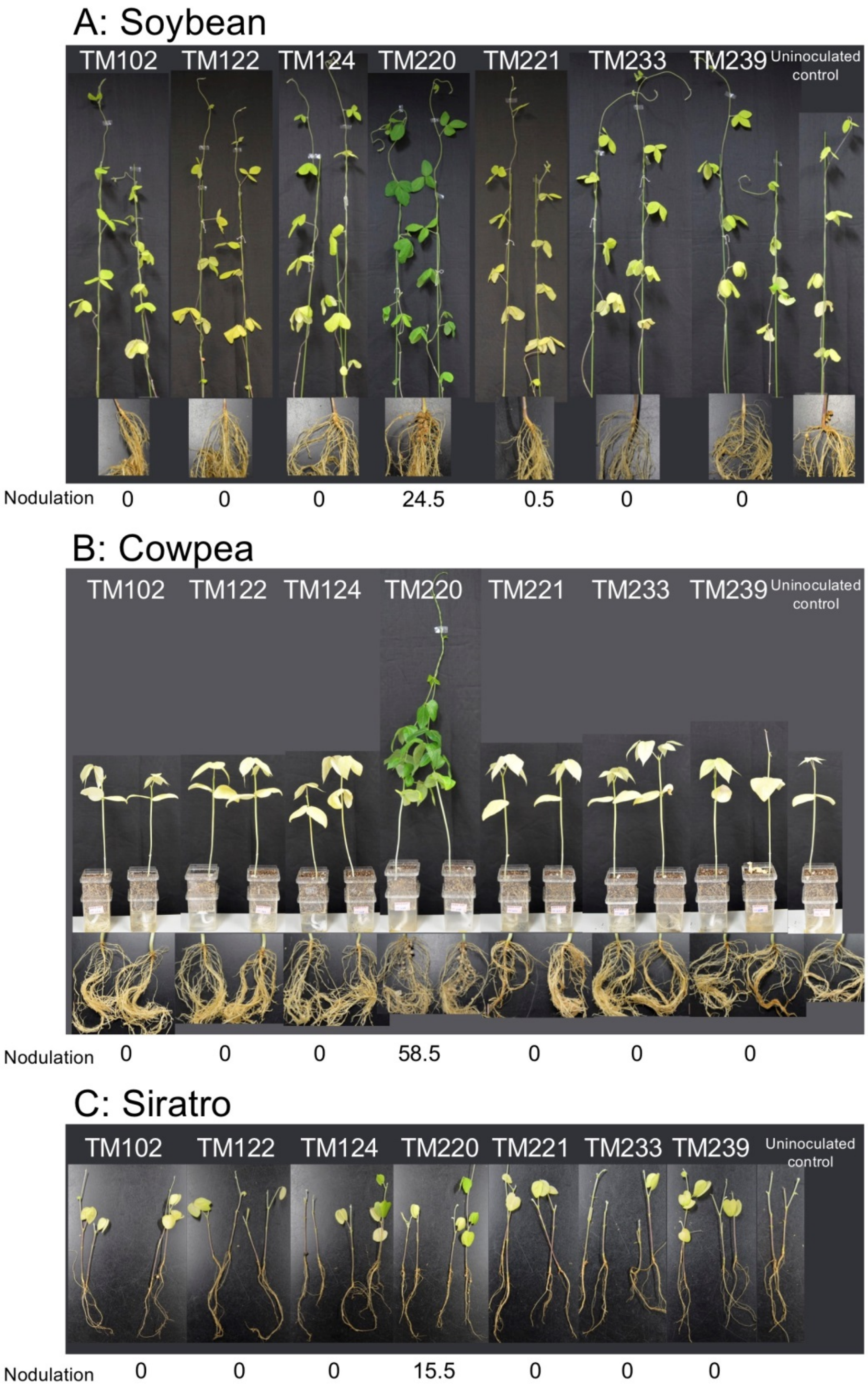

Fig. S3. Nodulation of sorghum bradyrhizobial isolates with three leguminous plants of soybean (A), cowpea (B), and siratro (C). "Nodulation" denotes the average number of nodules plant^{-1} (n = 2~4). Acetylene reduction activity (ARA) for the N_2 fixation of soybean nodules formed with *Bradyrhizobium* sp. TM220 (A) exhibited 24.3 µmol h^{-1} plant^{-1} (29.0 µmol h^{-1} g nodule fresh weight^{-1}).

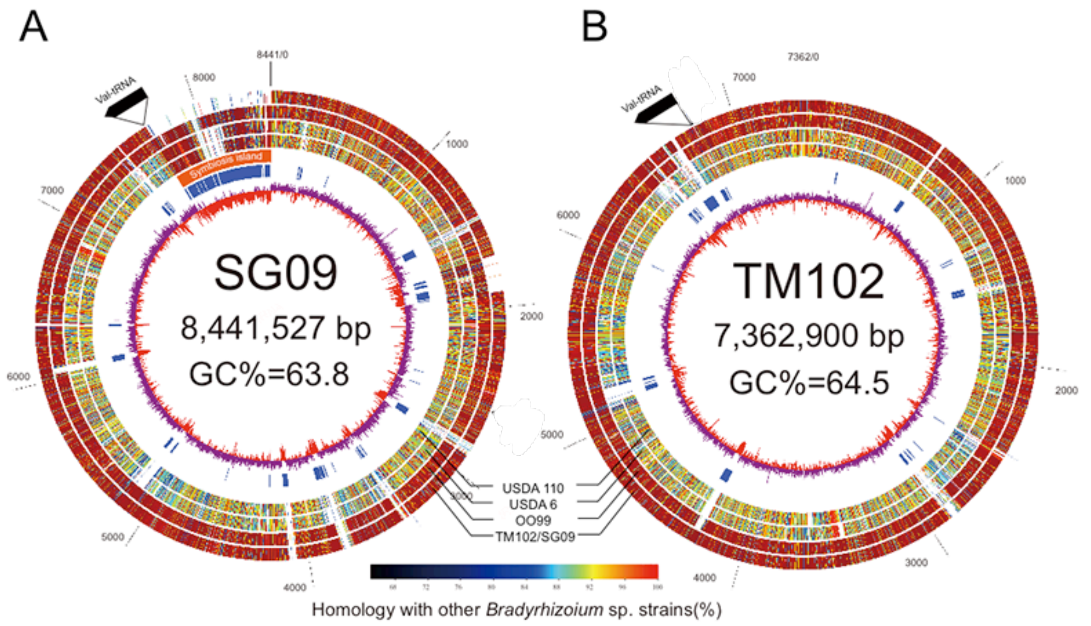

Fig. S4. The complete genomes of two *Bradyrhizobium* isolates SG09 (A) and TM102 (B) of group W (*B. ottawaense*), and MiSeq read mapping on the SG09 genome (C). (AB) The innermost circle shows the GC content (red and purple indicates a value lower and higher than the threshold, respectively). The blue bars in the second inner circle denote the genomic islands, as predicted by SIGI-HMM and IslandPath-DIMOB. The orange bar in the third inner circle in strain SG09 denote the symbiosis island. The inner fourth to sixth circles indicate the BlastN homology (bl2seq, calculated by GenomeMatcher (1)) with four strains of *Bradyrhizobium*: *B. diazoefficiens* USDA110^T, *B. japonicum* USDA6^T, and *B. ottawaense* OO99^T from the inside to the outside. The outmost circles indicate the BlastN homology with TM102 on the SG09 genome (A) and with G1-9 on the TM102 genome (B). The black arrow indicates the position of the Val-tRNA gene that was a target sequence of the symbiosis island of soybean bradyrhizobia (2, 3).

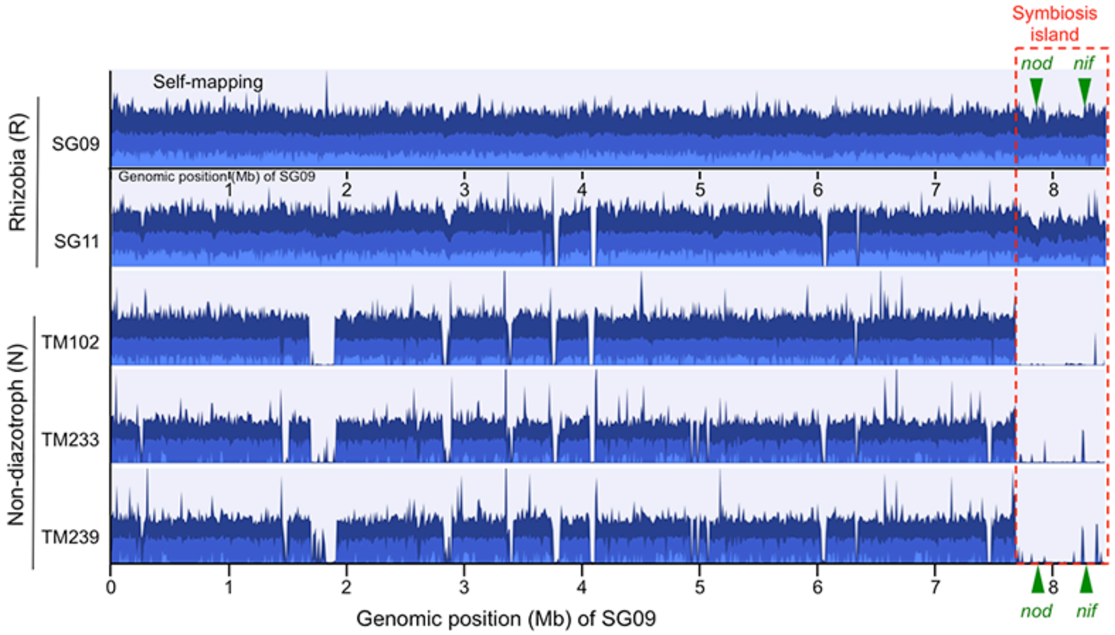

Fig. S5. Mapping of group W members on SG09 genome. The positions of the *nod* and *nif* gene clusters are indicated as green arrowheads within the symbiosis island of the SG09 genome. The mapping was carried out as described previously (4).

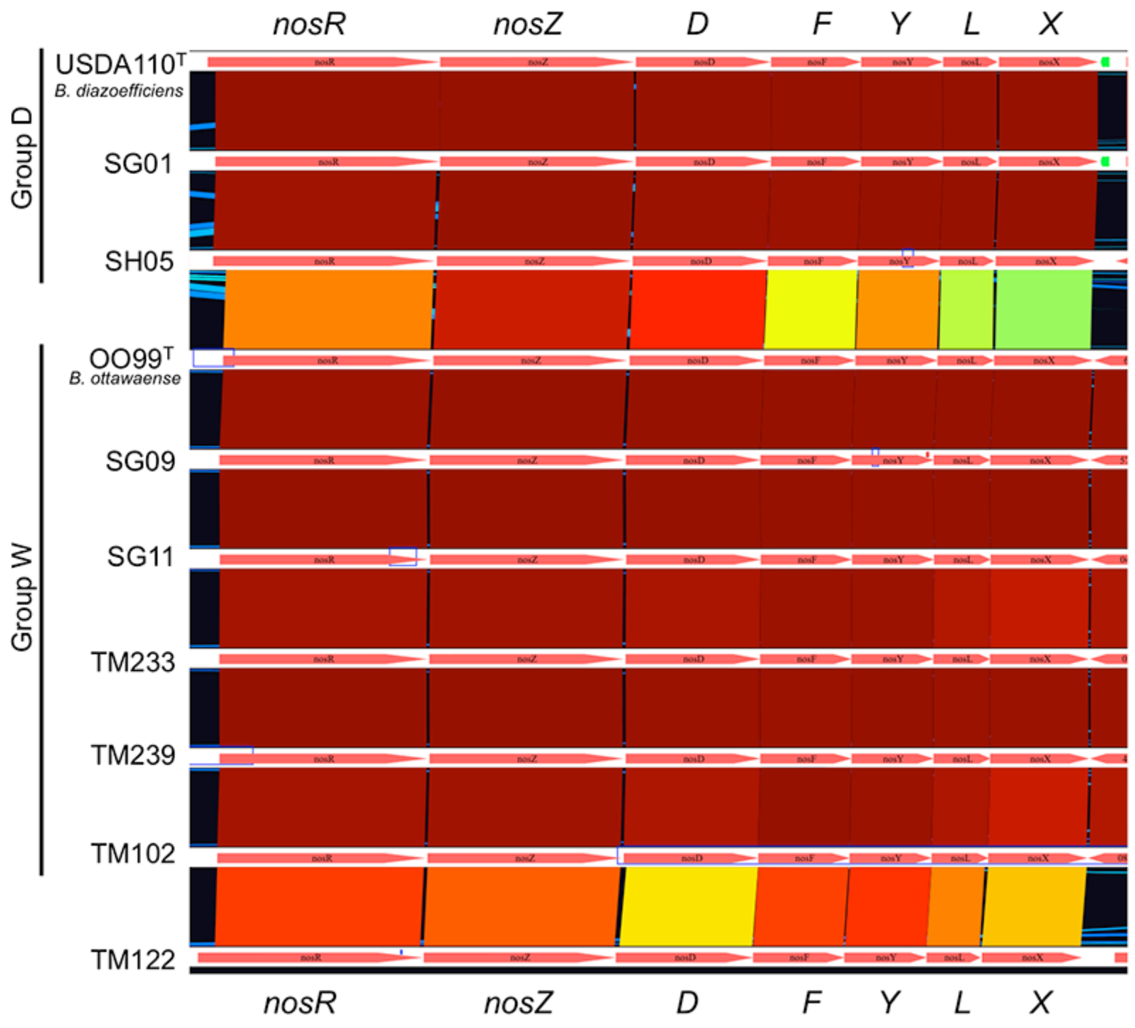

Fig. S6. Comparison of *nos* gene clusters in bradyrhizobial isolates of groups D and W members and TM122 based on amino acid sequences.

References

1. Ohtsubo, Y., W. Ikeda-Ohtsubo, Y. Nagata, and M. Tsuda. 2008. GenomeMatcher: A graphical user interface for DNA sequence comparison. BMC Bioinf. 9:376.
2. Kaneko, T., Y. Nakamura, S. Sato, *et al.* 2002. Complete genomic sequence of nitrogen-fixing symbiotic bacterium *Bradyrhizobium japonicum* USDA110. DNA Research. 9:189-197.
3. Kaneko, T., H. Maita, H. Hirakawa, *et al.* 2011. Complete genome sequence of the soybean symbiont *Bradyrhizobium japonicum* strain USDA6^T. Genes 2:763-787.
4. Hara, S, T. Morikawa, S. Wasai, Y. Kasahara, T. Koshiba, K. Yamazaki, T. Fujiwara, T. Tokunaga, and K. Minamisawa. 2019. Identification of nitrogen-fixing *Bradyrhizobium* associated with roots of field-grown sorghum by metagenome and proteome analyses. Front. Microbiol. 10:407.

Table S1. List of bradyrhizobial isolats from field-grown sorghum roots

Strain	Sorghum line[*1]	Plot[*2]	Isolation	OTU (97%)	Selected isolates	ARA (nmol C_2H_4 $tube^{-1}$ h^{-1})[*3]	Best hit of ITS[*4]	ITS accession	Draft genome
SG01	KM2	Plot 1	Trapping	OTU 4	○	< 0.01	*B. diazoefficiens* 110spc4	LC494456	DRX177056
SG02	KM2	Plot 1	Trapping	OTU 4		< 0.01	*B. diazoefficiens* USDA122	LC494457	
SG03	KM2	Plot 1	Trapping	OTU 4	○	< 0.01	*B. diazoefficiens* 110spc4	LC494458	DRX177057
SG04	KM2	Plot 1	Trapping	OTU 4		< 0.01	*B. diazoefficiens* USDA122	LC494459	
SG05	KM2	Plot 1	Trapping	OTU 4		< 0.01	*B. diazoefficiens* NK6	LC494460	
SG06	KM2	Plot 1	Trapping	OTU 4		< 0.01	*B. diazoefficiens* NK6	LC494461	
SG07	KM2	Plot 1	Trapping	OTU 4		< 0.01	*B. diazoefficiens* USDA122	LC494462	
SG08	KM2	Plot 1	Trapping	OTU 4		< 0.01	*B. diazoefficiens* 110spc4	LC494463	
SG09	KM2	Plot 1	Trapping	OTU 5	○	< 0.01	*B. ottawaense* OO99	LC494464	DRX177058
SG10	KM2	Plot 1	Trapping	OTU 5		< 0.01	*B. ottawaense* OO99	LC494465	
SG11	KM2	Plot 1	Trapping	OTU 5	○	< 0.01	*B. ottawaense* OO99	LC494466	DRX177059
SG12	KM2	Plot 1	Trapping	OTU 4		< 0.01	*B. diazoefficiens* USDA122	LC494467	
SG13	KM2	Plot 1	Trapping	OTU 4		< 0.01	*B. diazoefficiens* NK6	LC494468	
SG14	KM2	Plot 1	Trapping	OTU 4		< 0.01	*B. diazoefficiens* USDA122	LC494469	
SG15	KM2	Plot 1	Trapping	OTU 4	○	< 0.01	*B. diazoefficiens* USDA122	LC494470	DRX177060
SG16	KM2	Plot 1	Trapping	OTU 4		< 0.01	*B. diazoefficiens* USDA122	LC494471	
SG17	KM2	Plot 1	Trapping	OTU 4		< 0.01	*B. diazoefficiens* USDA122	LC494472	
SG18	KM2	Plot 1	Trapping	OTU 1	○	< 0.01	*Bradyrhizobium* sp. WB66	LC494473	DRX177061
SG19	KM2	Plot 1	Trapping	OTU 4		< 0.01	*B. diazoefficiens* 110spc4	LC494474	
SG20	KM2	Plot 1	Trapping	OTU 5		< 0.01	*B. ottawaense* OO99	LC494475	
SG21	KM2	Plot 1	Trapping	OTU 4		< 0.01	*B. diazoefficiens* USDA122	LC494476	
SG22	KM2	Plot 1	Trapping	OTU 2		< 0.01	*B. japonicum* T7	LC494477	
SG23	KM2	Plot 1	Trapping	OTU 5		< 0.01	*B. ottawaense* OO99	LC494478	
SG24	KM2	Plot 1	Trapping	OTU 4		< 0.01	*B. diazoefficiens* USDA122	LC494479	
SG25	KM2	Plot 1	Trapping	OTU 4		< 0.01	*B. diazoefficiens* USDA122	LC494480	
SG26	KM2	Plot 1	Trapping	OTU 4		< 0.01	*B. diazoefficiens* 110spc4	LC494481	
SG27	KM2	Plot 1	Trapping	OTU 4		< 0.01	*B. diazoefficiens* USDA122	LC494482	
SG28	KM2	Plot 1	Trapping	OTU 2	○	< 0.01	*B. japonicum* J5	LC494483	DRX177062
SG29	KM2	Plot 1	Trapping	OTU 2		< 0.01	*B. japonicum* J5	LC494484	
SH03	KM2	Plot 2	Trapping	OTU 1	○	< 0.01	*B. japonicum* USDA38	LC494485	DRX177063
SH05	KM2	Plot 2	Trapping	OTU 4	○	< 0.01	*B. diazoefficiens* USDA122	LC494486	DRX177064
SH10	KM2	Plot 2	Trapping	OTU 4	○	< 0.01	*B. diazoefficiens* NK6	LC494487	DRX177065
SH11	KM2	Plot 2	Trapping	OTU 4	○	< 0.01	*B. diazoefficiens* NK6	LC494488	DRX177066
SH21	KM2	Plot 2	Trapping	OTU 2	○	< 0.01	*B. japonicum* 05LoS24R2.25	LC494489	DRX177067
SH24	KM2	Plot 2	Trapping	OTU 4	○	< 0.01	*B. diazoefficiens* NK6	LC494490	DRX177068
SF01	KM2	Plot 3	Trapping	OTU 1	○	< 0.01	*B. japonicum* USDA32	LC494491	DRX177069
SF05	KM2	Plot 3	Trapping	OTU 4	○	< 0.01	*B. diazoefficiens* NK6	LC494492	DRX177070
SF11	KM2	Plot 3	Trapping	OTU 1	○	< 0.01	*B. japonicum* 953	LC494493	DRX177071
TM102	KM1	Plot 1	Direct	OTU 5	○	< 0.01	*B. ottawaense* OO99	LC495155	DRX177072
TM122	KM2	Plot 1	Direct		○	1.02	*Bradyrhizobium* sp. S58		DRA006492
TM124	KM2	Plot 1	Direct	OTU 6	○	18.3	*Bradyrhizobium* sp. S23321	LC495156	DRA006493
TM220	KM1	Plot 1	Direct	OTU 4	○	< 0.01	*B. diazoefficiens* 110spc4	LC495153	DRX177052
TM221	KM1	Plot 1	Direct	OTU 3	○	11	*B. diazoefficiens* NK6	LC495154	DRX177053
TM233	KM2	Plot 1	Direct	OTU 5	○	< 0.01	*B. ottawaense* OO99	LC495152	DRX177054
TM239	KM2	Plot 1	Direct	OTU 5	○	< 0.01	*B. ottawaense* OO99	LC495151	DRX177055

[*1] We used two sorghum lines described in previous paper (Hara *et al.* 2019).

[*2] We planted each line in three plots described in detail in previous paper (Hara *et al.* 2019).

[*3] Average of ARA (Acetylene-reducing activity) with three independent test tubes. Detection limit is 0.01 nmol C_2H_4 h^{-1} $tube^{-1}$.

[*4] Best hit in the results of NCBI BLAST based on ITS region DNA sequence (https://blast.ncbi.nlm.nih.gov/Blast.cgi).

Hara, S, T. Morikawa, S. Wasai, Y. Kasahara, T. Koshiba, K. Yamazaki, T. Fujiwara, T. Tokunaga, and K. Minamisawa. 2019. Identification of nitrogen-fixing *Bradyrhizobium* associated with roots of field-grown sorghum by metagenome and proteome analyses.

Table S2. General features of SG09 and TM102 genomes that were determined in the present work

	SG09	TM102	OO99^T	USDA110^T	USDA6^T	S58^T	S23321
Accession no.	AP021854	AP021855	CP029425	BA000040	AP012206	AP012603	AP012279
Size (bp)	8,441,527	7,362,900	8,606,328	9,105,828	9,207,384	8,264,165	7,231,841
GC content (%)	63.8	64.5	63.8	64.1	63.7	65.1	64.3
Size of symbiosis island (kb)	759	None	527	681	694-699	None	None
tRNA coding genes*	57	56	55	56	59	54	51
rRNA gene cluster*	1	1	1	1	2	2	1
CDS*	8,047	6,955	8,062	8,620	8,829	7,214	6,892
Gene density (bp)	1,049	1,059	1,067	1,056	1,043	1146	1,049

*Number of tRNA, rRNA and CDS was calculated by DFAST (https://dfast.nig.ac.jp/).
